# Pan-cancer analysis of TASL: a novel immune infiltration-related biomarker for tumor prognosis and immunotherapy response prediction

**DOI:** 10.1186/s12885-023-11015-w

**Published:** 2023-06-09

**Authors:** Huanyu Li, Xiaoyu Sun, Yanyun Zhao, Changzhu Zhang, Kai Jiang, Jie Ren, Lijuan Xing, Miao He

**Affiliations:** 1 Department of Pharmacology, School of Pharmacy, China Medical University, Shenyang, 110122 Liaoning Province China; 2 Liaoning Key Laboratory of Molecular Targeted Anti-Tumor Drug Development and Evaluation, Key Laboratory of Precision Diagnosis and Treatment of GastrointestinalTumors (China Medical University), Ministry of Education, Liaoning Cancer Immune Peptide Drug Engineering Technology Research Center, Shenyang, 110122 Liaoning Province China; 3 Precision Laboratory, Panjin Central Hospital, Panjin, 124000 Liaoning Province China

**Keywords:** TASL, Toll-like receptor, Tumor immunotherapy, Tumor microenvironment, Biomarker

## Abstract

**Background:**

New immunotherapeutic strategies based on predictors are urgently needed. Toll-like receptor adaptor interacting with SLC15A4 on the lysosome (TASL) was recently confirmed to fulfill an important role in the innate immune response. However, whether TASL is involved in tumor development and immunotherapy response prediction has not been reported.

**Methods:**

TCGA and GTEx were used to yield transcriptional, genetic, and epigenetic levels of TASL in 33 cancer types. CIBERSORT was used to explore the correlation between TASL expression and multiple immune-related signatures and tumor-infiltrating immune cell content in different cancer types. The ability of TASL to predict tumor immunotherapy response was analyzed in seven datasets. Finally, we tested TASL expression in human glioma cell lines and tissue samples and analyzed its correlation with clinicopathological parameters.

**Results:**

TASL is widely heterogeneous at the transcriptional, genetic, and epigenetic levels. High TASL expression is an independent poor prognostic factor for immune “cold” tumor Low-Grade Glioma (LGG) but an opposite factor for “hot” tumors Lung Adenocarcinoma (LUAD) and Skin Cutaneous Melanoma (SKCM). TASL may affect tumor immune infiltration by mediating tumor-infiltrating lymphocytes and tumor-associated macrophages. It may differentially affect the prognosis of the three cancers by regulating the immunosuppressive microenvironment in LGG and the immunostimulatory microenvironment in LUAD and SKCM. High TASL expression is a potential biomarker for the positive response to immunotherapy in cancers such as SKCM and was also experimentally confirmed to be positively associated with adverse clinicopathological features of gliomas.

**Conclusion:**

TASL expression is an independent prognostic factor for LGG, LUAD, and SKCM. High TASL expression is a potential biomarker for the positive response to immunotherapy in certain cancer types such as SKCM. Further basic studies focusing on TASL expression and tumor immunotherapy are urgently needed.

**Supplementary Information:**

The online version contains supplementary material available at 10.1186/s12885-023-11015-w.

## Introduction

Cancer treatment has undergone a radical change in recent years due to the use of immunotherapy [1]. Current cancer immunotherapy strategies, most of which target and enhance the acquired immune response, such as numerous immune checkpoint inhibitors (ICIs), have been used in the clinic to date [2]. However, the overall effectiveness of these therapies in patients is only about 20% [3]. In addition, only “hot” tumors with T-cell infiltration (such as skin cutaneous melanoma, non-small cell lung cancer, and kidney cancer [4]) had a durable clinical benefit, while “cold” tumors (such as gliomas [5, 6] and ER^+^ breast cancer [7]) have poor efficacy because of the absence of T-cell infiltration [8]. PD-L1 expression [9], tumor mutational burden (TMB) [10], as well as microsatellite instability or DNA mismatch repair deficiency (MSI/dMMR) [11] have been approved by the Food and Drug Administration (FDA) for predicting the efficacy of treatment with ICIs in cancer patients. However, they work only for a few cancer types [12, 13]. Therefore, finding novel tumor immunotherapy targets and predictive biomarkers for therapeutic response remains a daunting task.

Immunotherapy requires a coordinated approach to innate and adaptive immunity [14]. Release of tumor antigens to the killing of tumor cells by T cells requires the antigens processing and presentation by innate immune cells, and then, activation of adaptive immunity [15]. Recently several cancer therapies targeting innate immunity have emerged clinically to convert immune “cold” tumors into immune “hot” tumors [14], including the activation of pattern recognition receptors (PRRs), such as Toll-like receptors (TLRs). TLRs expressed on cancer cells and immune cells have a bidirectional regulation of the tumor microenvironment (TME). Activating TLRs on immune cells can trigger an intracellular signaling cascade involving adopters, inflammatory cytokines, and chemokines through multiple mechanisms to activate intrinsic immune cells and clear tumor cells. Activation of TLR7 on gastric cancer cells triggers apoptosis [16], while activation of TLR7 on glioma cells and TLR4 on tumor cells triggers cell proliferation [17, 18]. TLR pathway activation-mediated broad immune stimulation opens up ideas for treating “cold” tumors. Currently, there are three TLRs agonists, BCG (TLR2/TLR4 agonist) [19], imiquimod (R-837) (TLR7 agonist) [20], and monophospholipid A (MPLA) (TLR4 agonist) [21] approved for bladder cancer, basal cell carcinoma, and vaccine adjuvant, respectively.

Notably, most TLRs require downstream adaptors to initiate signaling. TASL (TLR adaptor interacting with SLC15A4 on the lysosome), encoded by the *TASL* (alias *CXorf21*) gene, is associated with systemic lupus erythematosus (SLE) [22]. In 2020, Heinz LX et al. revealed for the first time an essential role of TASL in the innate immune response. TASL acts as an adaptor for endosomal lysosomal TLR7-9 signaling and binds to the soluble carrier protein 15a4 (SLC15A4) to compose a complex and specifically activates interferon (IFN) regulatory factor 5 (IRF5) to mediate the downstream immune response [23]. But we do not know whether TASL is involved in tumor development and immunotherapy response prediction. Based on this, this study was conducted to explore the correlation between TASL and tumor prognosis, immune infiltration, and immunotherapy in 33 cancer types using databases such as the Cancer Genome Atlas (TCGA) dataset, the Genotype-Tissue Expression (GTEx) dataset as well as the Gene Expression Omnibus (GEO) dataset, to provide ideas for further research on the prognostic and therapeutic potential of TASL.

## Results

### TASL expression in different cancer types

**Fig. 1:**
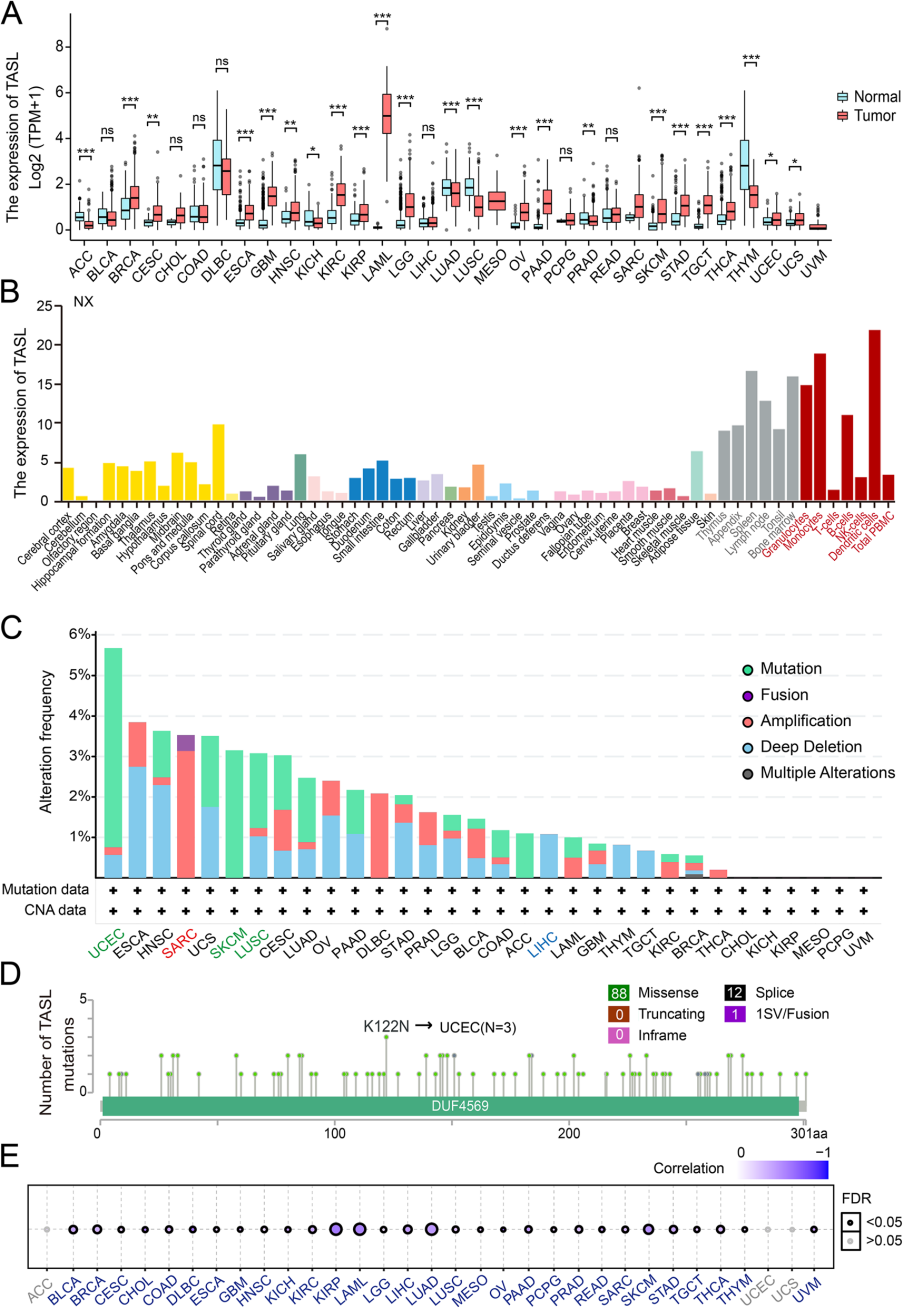
Overview of TASL mRNA expression and mutations in tumor and normal tissues. **A** TASL mRNA expression between tumor and normal tissues in TCGA and GTEx. **B** TASL mRNA expression profile in normal human tissues from HPA database. Types, frequency (**C**) and sites (**D**) of TASL mutations in various types of cancers from TCGA. **E** Spearman's correlation between TASL mRNA expression and DNA methylation in 33 kinds of cancers from TCGA, with larger circles representing smaller FDRs. (*P < 0.05; **P < 0.01; ***P < 0.001)

TASL mRNA is significantly differentially expressed in tumor and normal groups in 20 of the 33 kinds of cancers (*P* < 0.01, Fig. 1A). In general, TASL expression was upregulated in most types of tumor samples compared with normal samples, including BRCA, CESC, GBM, HNSC, LGG, OV, SKCM, et al., while downregulated in ACC, LUAD, LUSC, et al. This may suggest that TASL plays different roles in different tumor processes. In addition, we assessed TASL mRNA expression in various normal tissues using the HPA database and found that TASL was enriched in blood, bone marrow, and lymphoid tissues where immune cells were accumulated (Fig. 1B).

Genetic variants contribute to cancer progression and immune escape. In addition, the cBioPortal platform was utilized to explore the genetic variation in TASL in the TCGA cohort of cancer patients. TASL has the highest mutation frequency in UCEC (~ 5%), followed by SKCM (~ 3%) and LUSC (~ 2%). CNA “amplified” type was the main type of genetic variation in SARC, > 3%), and all LIHC with genetic variation had TASL copy number deletion (~ 1%, Fig. 1C). The mutation sites and number of TASL are further shown in Fig. 1D, we found that missense mutations (88 cases) in TASL were the main type of genetic alteration, and K122N was the most common mutation site, detected in three cases of UCEC. We also performed an analysis of the correlation between mRNA expression and DNA methylation of TASL and found a negative correlation between the two in 30 cancer types including KIRP, LAML, and LUAD (FDR < 0.05, Fig. 1E). In conclusion, we found extensive heterogeneity in TASL of human cancers at the transcriptional, genetic and epigenetic levels.

### TASL expression and immunotherapy

#### TASL is significantly associated with the immune infiltration signatures of 20 cancer types

**Fig. 2:**
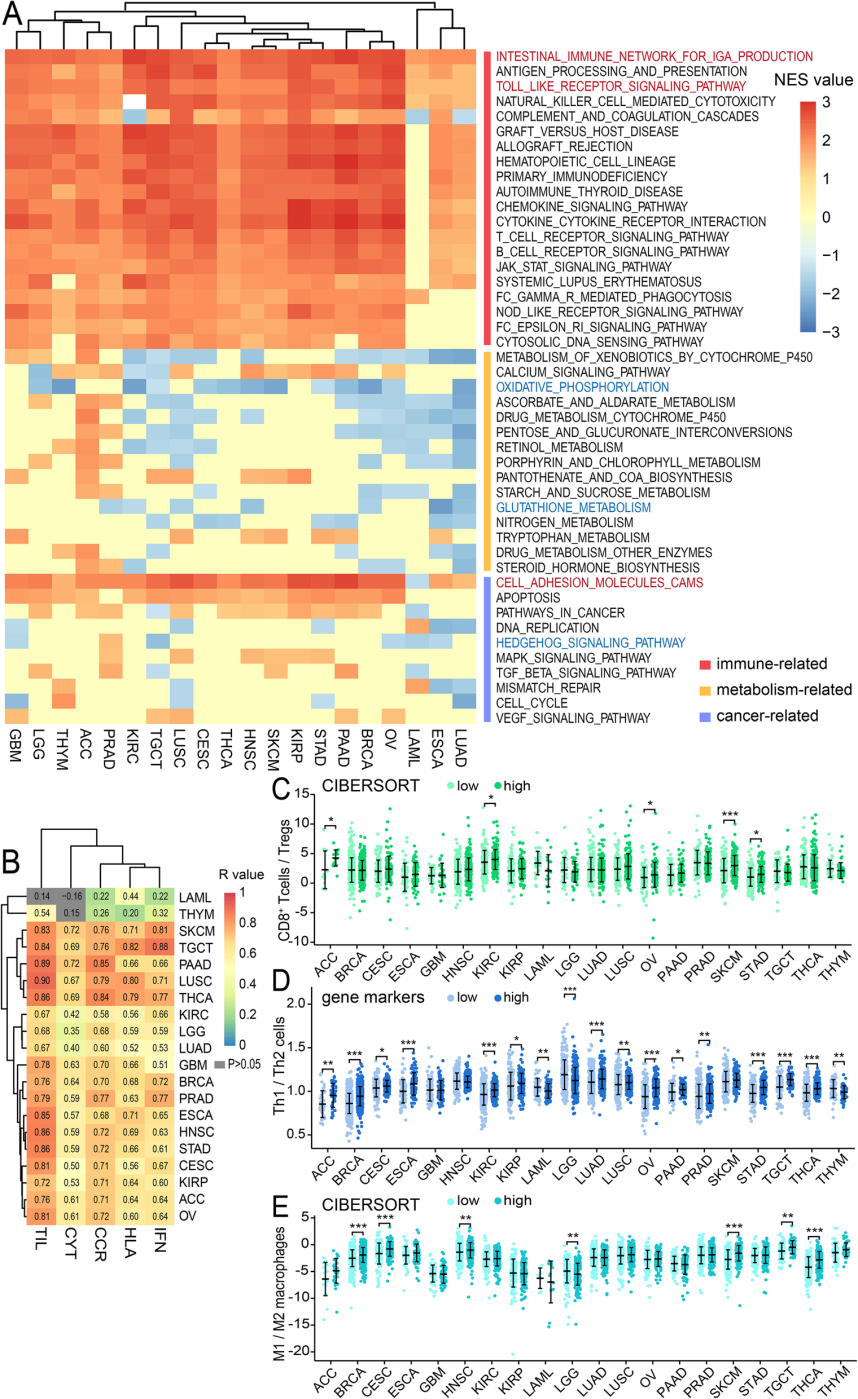
Correlation of TASL expression with immune infiltration signatures. **A** Relationship between TASL expression and KEGG pathways in 20 cancer types obtained by GSEA. **B** Spearman’s correlation of TASL expression with CYT, CCR, HLA, TIL and IFN signatures. The ratios of CD8^+^ T cells to Tregs (**C**), Th1 cells to Th2 cells (**D**), and M1 macrophages to M2 macrophages (**E**) in the TASL low and high expression groups from 20 types of cancers of TCGA (*P < 0.05; **P < 0.01; ***P < 0.001)

Firstly, we divided patients into two groups, “high” and “low”, based on the median value of TASL expression in the TCGA's 20 cancer cohorts, and then performed GSEA for KEGG pathways. The clustering heatmap showed 20 immune-related pathways, 15 metabolism-related pathways, and 10 cancer-related pathways that were significantly enriched (|NES|≥ 1.0, *P* < 0.05, FDR < 0.25, Fig. 2A). The results showed that several immune-related pathways such as “intestinal immune network for IgA production”, “Toll-like receptor signaling pathway”, etc., were significantly upregulated in the TASL high expression group in most cancer types. Interestingly, we also found that most of the significantly enriched metabolic pathways were widely downregulated in the TASL high expression group of 20 types of cancers, such as “oxidative phosphorylation”, and “glutathione metabolism”, etc. In addition, TASL also affected several cancer-related pathways, including “cell adhesion molecule CAMs”, “hedgehog signaling pathway”, etc. These data suggest that TASL may influence cancer progression by regulating immunity, energy metabolism, and cell proliferation and metastasis, detailed information is shown in Supplementary Table 1.

We used multiple immune infiltration signatures, such as CYT, CCR, HLA, TIL, and IFN, to assess the relationship between TASL expression and tumor immunity. We found that TASL expression was highly positively correlated with these immune signatures in 20 cancer types, in particular, the correlation between TIL and TASL expression was most significant (Fig. 2B, Supplementary Table 2). Based on these, we divided each type of cancer tissue into “high” and “low” groups according to the median value of TASL expression and assessed the infiltration levels of CD8^+^ T cells (immunostimulatory cells) and Tregs (immunosuppressive cells) using the CIBERSORT algorithm. The results showed that the ratio of CD8^+^ T cells to Tregs was significantly higher in the high group than in low group in ACC, KIRC, OV, SKCM, and STAD (Mann–Whitney U-test, *P* < 0.05, Fig. 2C). In addition, we also found that the ratio of Th1 (immunostimulatory cells) to Th2 (immunosuppressive cells) in the high group was significantly higher in ACC, BRCA, CESC, ESCA, KIRC, KIRP, LUAD, LUSC, OV, PAAD, PRAD, STAD, TGCT and THCA using the ssGSEA algorithm, while significantly lower in LAML, LGG and THYM. (Mann–Whitney U-test, *P* < 0.05, Fig. 2D).

Thereafter, we also evaluated the levels of M1 macrophages (immunostimulatory cells) and M2 macrophages (immunosuppressive cells) for each cancer type using CIBERSORT and calculated the ratios. Only the M1/M2 macrophage ratio of LGG was found to be significantly lower in the high subgroup, while the ratios of six other types of cancers (BRCA, CESC, HNSC, SKCM, TGCT and THCA) were significantly higher in the high subgroup (Mann–Whitney U-test, *P* < 0.05, Fig. 2E), indicating that TASL may affect tumor immunity mainly by mediating TILs and tumor-associated macrophages (TAMs).

#### TASL expression can more accurately predict the immune infiltration signatures of 20 cancer types

Since a high level of immune infiltration is a favorable condition affecting tumor response to immunotherapy [24, 25], we explored whether high TASL expression could predict a high level of immune infiltration in tumors. We divided patients into high and low groups based on the median immune score and TIL score (two indicators of immune infiltration) for each cancer cohort and the area under the curve (AUC) of the receiver operating characteristic (ROC) curves were used to assess the ability of TASL expression to predict the two indicators. TMB, MSI, and PD-L1 expression have been approved by the FDA for predicting the efficacy of ICIs in cancer patients, while PD-1 [26], CTLA-4 expression [27] and glycolysis score [28] have been reported as potential biomarkers for predicting response to ICIs therapy. Thus, we compared the predictive ability of TASL expression and these indexes on the level of tumor immune infiltration.

**Fig. 3:**
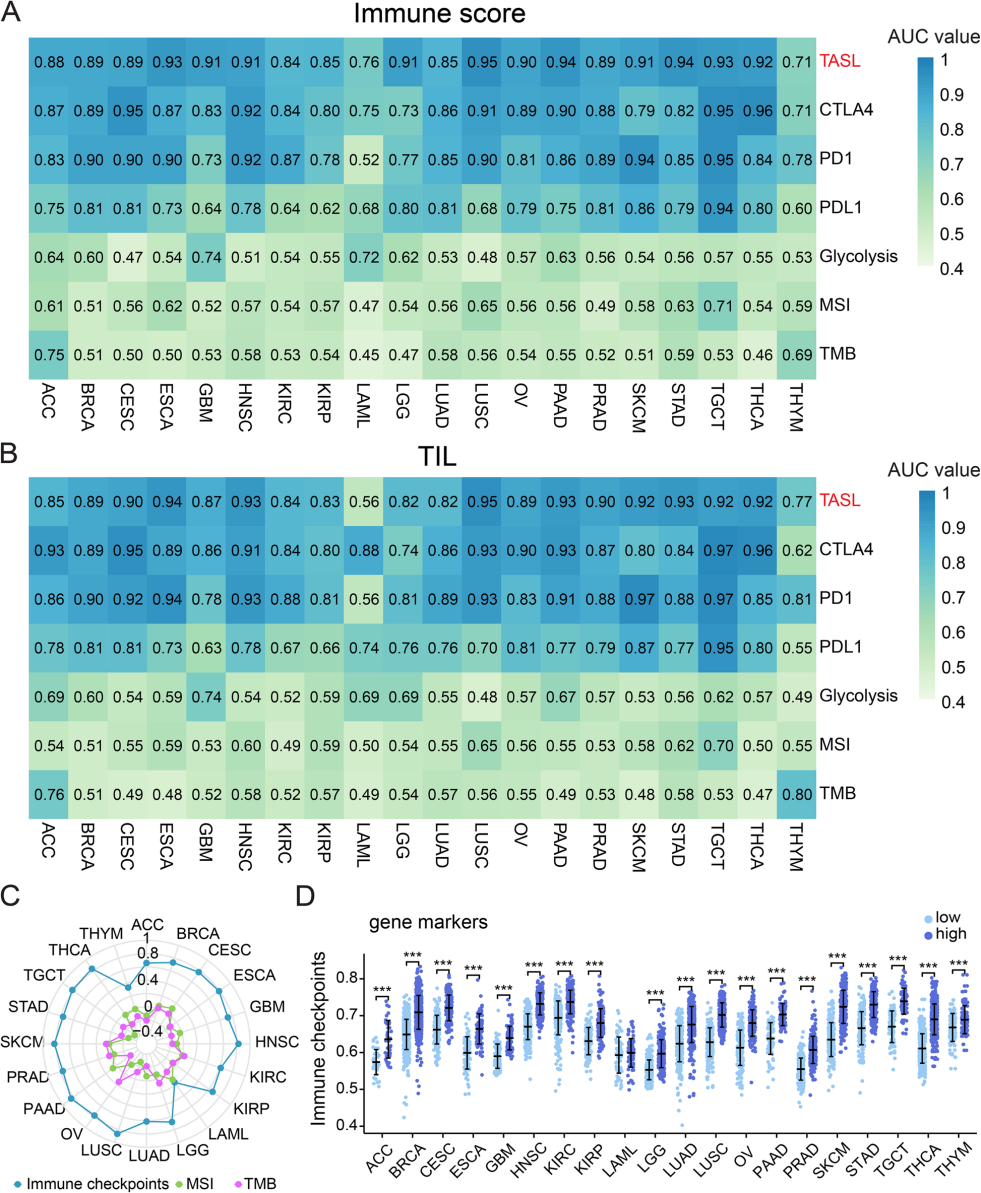
Predictive value of TASL expression on the immune signature of 20 cancer types. **A**-**B** AUC value of predicted immune score and TIL using TASL, PD-L1, PD-1, CTLA-4 expression, TMB, MSI and glycolysis score. **C** Spearman’s correlation between TASL expression and immune checkpoint gene expression, TMB, and MSI in 20 types of cancers in TCGA. **D** Differential expression of immune checkpoint genes in the TASL low and high expression groups of 20 types of cancers from TCGA (*P < 0.05; **P < 0.01; ***P < 0.001)

The analysis showed that for predicting immune scores of 20 cancer types, TASL was found in 11 cancer types (ESCA, GBM, HNSC, LGG, LUSC, OV, PAAD, SKCM, STAD, TGCT, and THCA), and CTLA-4 in 6 cancer types (CESC, HNSC, LUSC, PAAD, TGCT and THCA) and PD-1 expression in 7 cancer types (BRCA, CSC, ESCA, HNSC, LUSC, SKCM and TGCT) with high accuracy (AUC > 0.9). In contrast, PD-L1 expression had moderate potential (0.9 > AUC > 0.7) to predict immune scores in most cancer types, while glycolysis score, TMB and MSI performed poorly in predicting immune score accuracy (AUC, 0.45–0.75; Fig. 3A, Supplementary Table 3). Similarly, for TIL, TASL, PD-1, and CTLA-4 expression were highly accurate in 10, 8, and 8 cancer types, respectively. While in most cancer types, PD-L1 expression had moderate potential to predict TIL score, and glycolysis score, TMB and MSI had low accuracy in predicting TIL score (Fig. 3B, Supplementary Table 4). These results indicate that TASL expression is a stronger predictor of immune infiltration signatures.

Given the good predictive efficacy of TASL expression for immune infiltration, we further investigated the correlation between TASL expression and TMB, MSI, and immune checkpoint gene expression (calculated by ssGSEA) of 20 cancer types in TCGA. We observed that TASL expression was strongly positively correlated with immune checkpoint genes in 18 cancer types, except LAML and THYM, which were weakly positively correlated (*P* < 0.001, Fig. 3C). In contrast, there was a weak negative correlation between TASL expression and TMB of GBM, HNSC, LUAD, LUSC, OV, PAAD, STAD and THCA, and a weak negative correlation between TASL and MSI of HNSC, LUSC, OV, STAD and TGCT (*P* < 0.05, Fig. 3C). We also found that the patients in the high TASL expression group had a higher level of immune checkpoint gene expression (Fig. 3D). Since overexpression of immune checkpoint genes tends to be positively correlated with high response rates to immunotherapy [9], our analysis implied that TASL expression may be associated with tumor immunotherapy response.

#### TASL expression can be a potential predictor of positive response to immunotherapy in patients with tumors such as SKCM

We used 7 tumor immunotherapy datasets (GSE19423, GSE111636, GSE67501, Miao D et al. [29], GSE100797, GSE91061 and GSE121810) to investigate the correlation between TASL expression and response to tumor immunotherapy. The analysis involved BLCA, KIRC, Renal Cell Carcinoma(RCC), and SKCM, which were the first tumor immunotherapy applications, and GBM, which is considered an immune “cold” tumor. The treatments included BCG therapy, anti-PD-1 therapy, anti-CTLA-4 therapy, and adoptive T cell therapy, and all samples were obtained prior to immunotherapy (except the neoadjuvant anti-PD-1 with postoperative adjuvant anti-PD-1 (NA-aPD1) subgroup in the GBM dataset, which was obtained during surgery), the details of the patient cohort are listed in Table 1.

**Table 1:** Datasets and criteria for evaluating patients’ responses to immunotherapy for different cancer types

Data name	Cancer type	Therapeutic agent	Classified as “response”	Classified as “non-response”	Protocol
GSE19423	BLCA	BCG	responder(*n* = 43)	progressor(*n* = 5)	Cystoscopy and urinary cytology
GSE111636	BLCA	anti-PD-1(pembrolizumab)	responder(*n* = 6)	progressor(*n* = 5)	——
GSE67501	RCC	anti-PD-1(nivolumab)	‘CR’,‘PR’(*n* = 4)	‘SD’, ‘NR’(*n* = 7)	RECIST^a^
Miao D, et al.(aPD1)	KIRC	anti-PD-1(nivolumab)	‘CR’,‘PR’(*n* = 3)	‘SD’, ‘PD’(*n* = 13)	RECIST
GSE100797	SKCM	Adoptive T cell therapy	‘CR’,‘PR’(*n* = 10)	‘SD’, ‘PD’(*n* = 15)	RECIST
GSE91061	SKCM	anti-PD-1(nivolumab) or anti-CTLA-4 progression + anti-PD-1(nivolumab,ipilimumab)	‘CR’,‘PR’(*n* = 10)	‘SD’, ‘PD’(*n* = 39)	RECIST
GSE91061 (aCTLA4 prog aPD1)	SKCM	anti-CTLA-4 progression + anti-PD-1(nivolumab,ipilimumab)	‘CR’,‘PR’(*n* = 4)	‘SD’, ‘PD’(*n* = 22)	RECIST
GSE121810(NA-aPD1)	GBM	Neoadjuvant anti-PD-1 + adjuvant anti-PD-1(pembrolizumab)	——	——	——
GSE121810(A-aPD1)	GBM	Adjuvant anti-PD-1(pembrolizumab)	——	——	——

^a^*RECIST* Response Evaluation Criteria in Solid Tumor

**Fig. 4:**
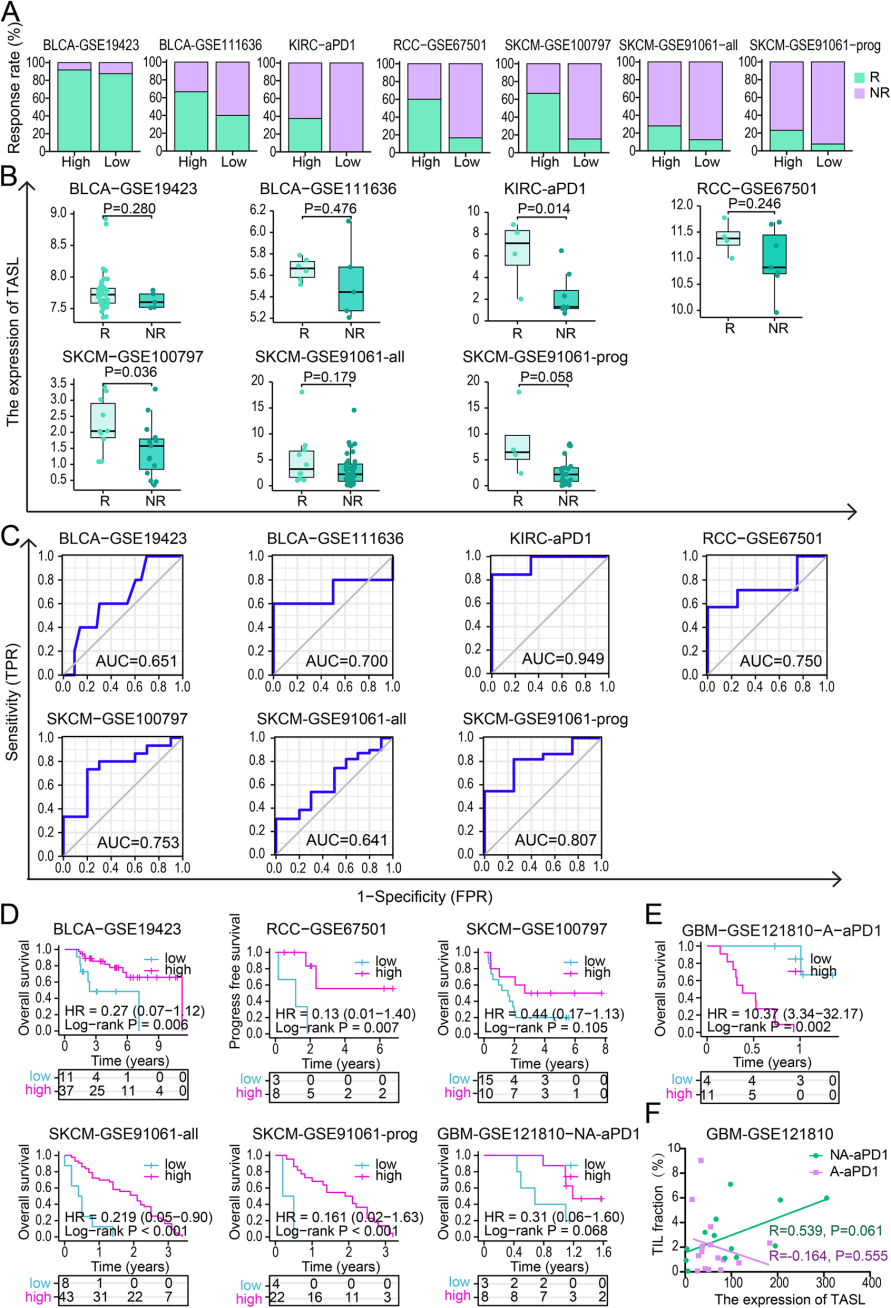
TASL expression is potentially predictive of immunotherapy response. **A** Response/non-response rates to treatment for low and high TASL expression groups in each immunotherapy dataset. **B** Differences in TASL expression between responders (R) and non-responders (NR). **C** ROC curves were used to assess the ability of TASL expression to differentiate responders (R) from non-responders (NR). **D**-**E** K-M curves of OS or PFS between low and high TASL expression groups. **F** Scatter plot of TIL fractions (%) and TASL expression in GSE121810 for group NA-aPD1 (green) and group A-aPD1 (purple), R: Spearman’s correlation coefficient

The analysis revealed that in BLCA, KIRC, RCC, and SKCM, the immunotherapy response rates were higher in the high TASL expression group (Fig. 4A), and the TASL expression levels were higher or marginally higher in immunotherapy responders (R) than in non-responders (NR) (KIRC (aPD1), *P* = 0.014; RCC-GSE67501, *P* = 0.246; SKCM-GSE100797, *P* = 0.036; SKCM-GSE91061-prog, *P* = 0.058; Fig. 4B). Next, we used ROC curves to assess the ability of TASL expression in differentiating responders from non-responders. The AUC values of BLCA-GSE111636, KIRC (aPD1), RCC-GSE67501, SKCM-GSE100797 and SKCM-GSE91061-prog reached 0.700, 0.949, 0.750, 0.753 and 0.807, respectively (Fig. 4C). This demonstrates that TASL expression has accuracy in predicting response to immunotherapy.

Analysis of clinical data revealed that TASL expression was positively correlated with longer OS or progress-free survival (PFS) in the immunotherapy-treated BLCA-GSE19423 (*P* = 0.006), RCC-GSE67501 (*P* = 0.007), SKCM-GSE91061-all (*P* < 0.001), SKCM-GSE91061-prog (*P* < 0.001) cohorts (Fig. 4D). As for the GSE121810 dataset of immune “cold” tumor, GBM, in the neoadjuvant anti-PD-1 with postoperative adjuvant anti-PD-1 (NA-aPD1) subgroup, patients in the TASL high expression group had a slightly longer OS (*P* = 0.068, Fig. 4D), whereas in the postoperative adjuvant anti-PD-1 (A-aPD1) subgroup, patients in the high TASL expression group had shorter OS (*P* = 0.002, Fig. 4E). GBM patients in the TASL high expression group without neoadjuvant anti-PD-1 therapy did not have a clinical benefit from postoperative anti-PD-1 therapy. To investigate the degree of immune cell infiltration at the tumor site in the two subgroups of patients, we further explored the relationship between TIL fraction (estimated by T cell receptor sequencing (TCR-seq)) and TASL expression in the tumors of both groups of patients and found that in the NA-aPD1 group, there was a positive trend of correlation between TIL fraction and TASL expression (*R* = 0.539, *P* = 0.061), whereas in the A-aPD1 group, TIL fraction was not associated with TASL expression (*R* = -0.164, *P* = 0.555, Fig. 4F), implying that in the NA-aPD1 subgroup, high TASL expression may be positively correlated with the activation of adaptive immunity. Taken together, the potential of TASL expression in predicting response to immunotherapy was further supported.

### TASL expression and prognosis

#### TASL expression is an independent prognostic factor in patients with LGG, LUAD and SKCM

**Fig. 5:**
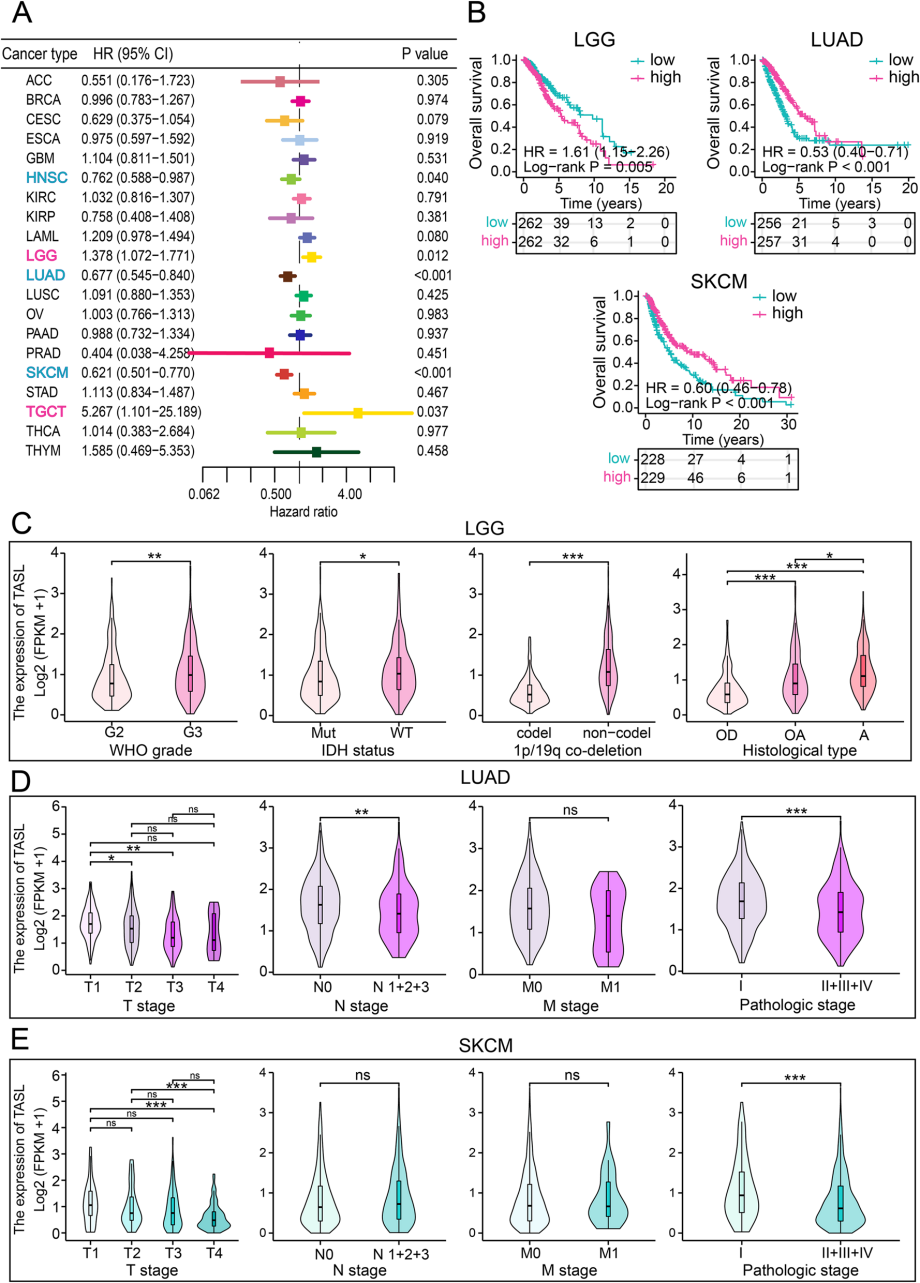
Association of TASL mRNA expression with OS and clinicopathological parameters in cancer patients. **A** Forest plot of Hazard Ratios of TASL for 20 cancers in TCGA. **B** K-M curves for OS of LGG, LUAD, and SKCM patients grouped by high and low TASL expression. **C** TASL expression in LGG patients with different WHO grades, IDH mutation status, 1p/19q co-deletion status, and pathological types. TASL expression in LUAD (**D**) and SKCM (**E**) with different AJCC TNM stages and pathological stages (*P < 0.05; **P < 0.01; ***P < 0.001)

COX regression analysis was to investigate the relationship between TASL expression and OS in patients with various cancers. Results showed that TASL expression was correlated with HNSC (HR, 0.762; 95% CI, 0.588–0.987; *P* = 0.040), LGG (HR, 1.378; 95% CI, 1.072–1.771; *P* = 0.012), LUAD (HR, 0.677; 95%CI, 0.455–0.840; *P* < 0.001), SKCM (HR, 0.621; 95%CI, 0.501–0.770; *P* < 0.001) and TGCT (HR, 5.267; 95%CI, 1.101–25.189; *P* = 0.037; Fig. 5A). Next, we divided tumor cases into two groups based on the median expression level of TASL. The K-M curves showed that the “cold” tumor LGG [6] patients with higher TASL expression tended to have an ominous prognosis (*P* < 0.01), while the “hot” tumors LUAD (*P* < 0.001) and SKCM [4] (*P* < 0.001) patients with higher TASL expression tended to have a better prognosis (Fig. 5B).

We also investigated the correlation between TASL expression and clinicopathological parameters in LGG, LUAD, and SKCM patients. Clearly, in LGG, TASL expression increased with the increase of WHO grades of patients (Fig. 5C). Inversely, TASL expression in LUAD and SKCM gradually decreased with the increase of TNM and pathological stage (Fig. 5D, E). The expression levels of TASL were higher in isocitrate dehydrogenase (IDH)-wildtype (IDH-WT) than that in IDH-mutant (IDH-Mut) patients, in 1p/19q non-co-deletion than that in 1p/19q co-deletion patients, and in astrocytomas (A) than that in oligoastrocytomas (OA) and oligodendrogliomas (OD; Fig. 5C). IDH-WT gliomas have a worse prognosis, and 1p/19q non-co-deletion gliomas are less sensitive to chemotherapy [30, 31]. These results suggested that TASL expression could help predict the prognosis of “cold” tumor LGG and “hot” tumors LUAD and SKCM.

**Fig. 6:**
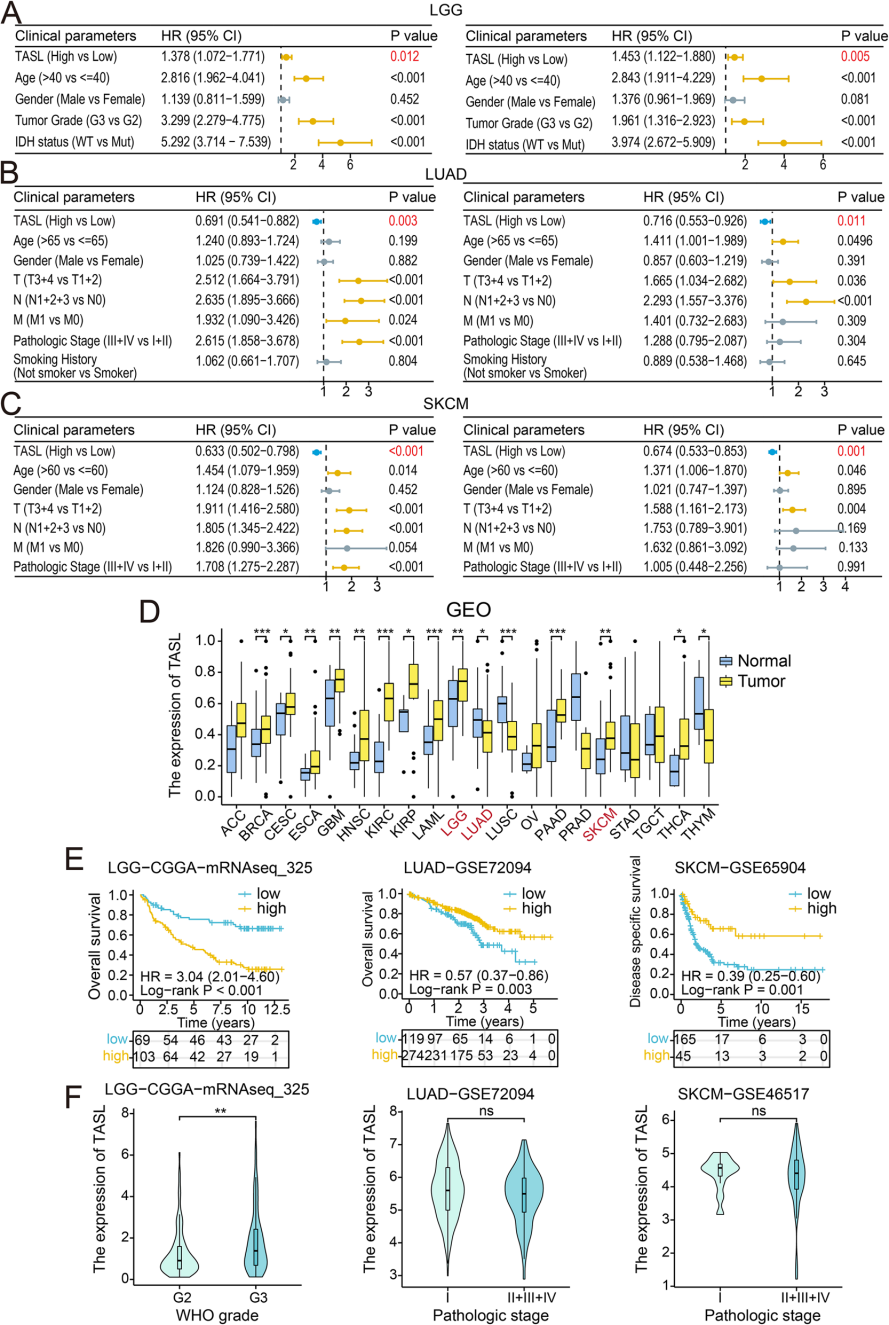
COX regression analysis of OS of LGG, LUAD, and SKCM patients in TCGA and validation in GEO and CGGA. Univariate (left) and multivariate (right) COX regression analysis of OS in LGG (**A**), LUAD (**B**), and SKCM (**C**) patients from TCGA. **D** Differences in TASL mRNA expression between tumor and normal tissues for 20 cancer types in GEO. (**E**) K-M curves for OS or DSS of LGG, LUAD, and SKCM patients grouped by high and low TASL expression from CGGA or GEO database. **F** Correlation of TASL expression with different WHO grades or pathological stages of LGG, LUAD, and SKCM (*P < 0.05; **P < 0.01; ***P < 0.001)

We further determine whether TASL expression can be served as an independent prognostic factor in patients with LGG, LUAD, and SKCM. The results showed that high TASL expression (HR, 1.453; 95%CI, 1.122 − 1.880; *P* = 0.005), age (HR, 2.843; 95%CI, 1.911 − 4.229;* P* < 0.001), high WHO grade (HR, 1.961; 95%CI, 1.316 − 2.923; *P* < 0.001) and IDH-Wild type (HR, 3.974; 95%CI, 2.672 − 5.909; *P* < 0.001) were significantly associated with shorter OS and could be considered as adverse independent factors affecting OS in LGG patients (Fig. 6A). While the high TASL expression in LUAD (HR, 0.716; 95%CI, 0.533–0.926; *P* = 0.011) and SKCM (HR, 0.674; 95%CI, 0.533–0.853; *P* = 0.001) was a good independent prognostic factor for OS (Fig. 6B, C). Next, we conducted a stratified survival analysis according to the clinical stage, and the best cut-off value of TASL expression was selected for grouping. The results showed that except for the G3 grade of LGG, T3 + T4, and M1 stage of LUAD, TASL expression had good predictive power for OS in patients with the three types of cancers in other clinical grades or stages (*P* < 0.05; Supplementary Fig. 1).

#### TASL expression is correlated with the different TIIC infiltration patterns in LGG, LUAD, and SKCM

**Fig. 7:**
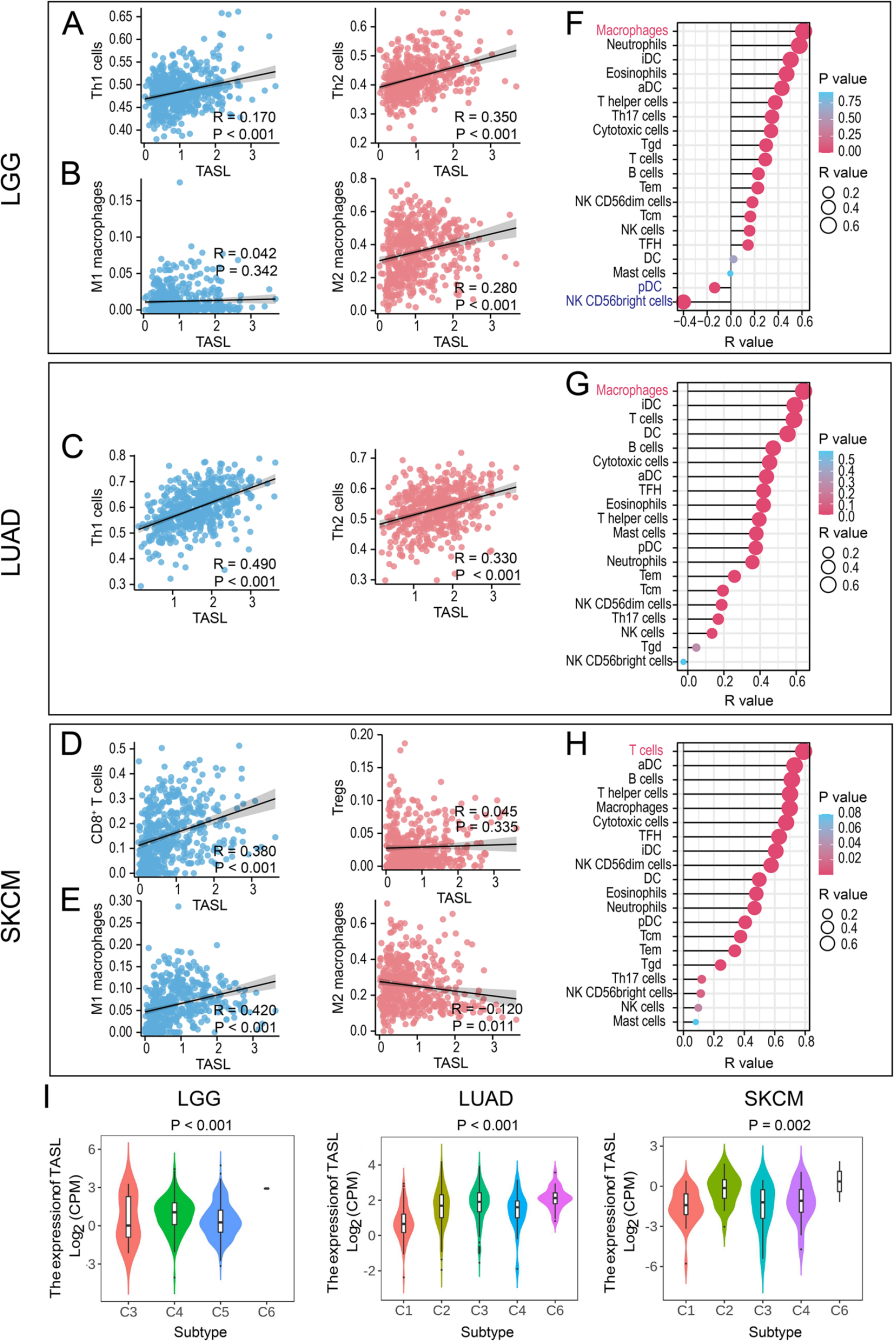
Relationship between TASL expression and the different TIIC patterns of LGG, LUAD, and SKCM. Correlation of TASL expression with Th1 cells, Th2 cells, CD8^+^ T cells, Tregs, M1 macrophages, or M2 macrophages content in LGG (**A**-**B**), LUAD (**C**) and SKCM (**D**-**E**). **F**–**H** Correlation of TASL expression with the infiltration level of 20 types of TIICs in TME of LGG, LUAD, and SKCM. **I** Expression of TASL mRNA in different immune subtypes of LGG, LUAD, and SKCM. R: Spearman’s correlation coefficient

Next, we analyzed the correlation between TASL expression and the content of immunostimulatory and immunosuppressive cells in the “cold” tumor LGG, the “hot” tumors LUAD and SKCM to further explore the relationship between TASL expression and various TIIC infiltration patterns. In the “cold” tumor LGG, TASL expression was significantly and positively correlated with the content of immunosuppressive Th2 cells (*R* = 0.350, *P* < 0.001) and M2 macrophages (*R* = 0.280, *P* < 0.001), and less correlated with the content of immunostimulatory Th1 cells (*R* = 0.170, *P* < 0.001, Fig. 7A, B). In the “hot” tumor LUAD, the correlation between TASL expression and immunostimulatory Th1 cells (*R* = 0.490, *P* < 0.001) was stronger than that of immunosuppressive Th2 cells (*R* = 0.330, *P* < 0.001, Fig. 7C). As for the “hot” tumor SKCM, TASL expression was positively correlated with the content of immunostimulatory CD8^+^ T cells (*R* = 0.380, *P* < 0.001) and M1 macrophages (*R* = 0.420, *P* < 0.001), while it was not correlated or negatively correlated with the content of immunosuppressive Tregs (*R* = 0.045, *P* = 0.335) and M2 macrophages (*R* = -0.120, *P* = 0.011, Fig. 7D, E).

We also used the ssGSEA algorithm to comprehensively estimate the infiltration levels of 20 other types of TIICs in the TME of LGG, LUAD, and SKCM, and calculated the correlation between TASL expression and the infiltration levels of TIICs. TASL expression was found to be significantly and positively correlated with the content of most TIICs in the three cancer types. In the “cold” tumor, LGG, TASL expression showed a significant positive correlation with macrophage (*R* = 0.614, *P* < 0.001) and a significant negative correlation with CD56^bright^ NK cells (*R* = -0.400, *P* < 0.001) and plasmacytoid dendritic cell (pDC) (*R* = -0.137, *P* = 0.002, Fig. 7F). In the “hot” tumor, LUAD, TASL expression most correlated with macrophages (*R* = 0.641, *P* < 0.001, Fig. 7G). In the “hot” tumor SKCM, TASL expression was positively correlated with all 20 types of TIICs, and the greatest correlation was found with T cells (*R* = 0.790, *P* < 0.001, Fig. 7H).

To verify our findings, we further studied the expression of TASL mRNA in different immune subtypes, which was a method of tumor typing proposed by Thorsson V et al. [32] in 2018, including C1 (wound healing); C2 (IFN-γ dominant); C3 (inflammatory); C4 (lymphocyte depleted); C5 (immunologically quiet) and C6 (TGF-β dominant). The highest expression levels of TASL were observed in the C4 subtype of LGG, the C6 subtype of LUAD, and the C2 subtype of SKCM (Kruskal–Wallis test, *P* < 0.01, Fig. 7I).

Considering the different relationship between TASL expression and survival of LGG, LUAD, and SKCM patients, the results of the analysis suggested that TASL may differentially affect the prognosis of the three cancer types by mediating an immunosuppressive microenvironment in “cold” tumor LGG and an immunostimulatory microenvironment in “hot” tumors LUAD and SKCM.

### Validation

#### Validation in GEO and CGGA database

To further verify the differences in TASL expression between tumor and normal samples, we compared microarray data for 20 types of cancers in the GEO database. Except for ACC, OV, PRAD, STAD, and TGCT, there were significant differences in TASL expression between other types of tumor and normal tissues (*P* < 0.05, Fig. 6D).

We also verified the predictive value of TASL expression on OS in LGG patients by analyzing the expression and clinical data of patients in the mRNAseq_325 cohort from the CGGA database. In the same way, LUAD patients from the GSE72094 dataset and SKCM patients from the GSE65904 dataset in the GEO database were included. Consistent with our results in the TCGA cohort, TASL expression was significantly associated with OS or disease-specific survival (DSS) in LGG (*P* < 0.001), LUAD (*P* = 0.003) and SKCM (*P* = 0.001, Fig. 6E). Violin plots showed that the median expression level of TASL in LGG patients in G3 was higher than that in G2 (*P* < 0.01), the median level of TASL expression in LUAD (*P* = 0.070) and SKCM (*P* = 0.665) patients with high pathological stage tended to be slightly lower than that in patients with low pathological stage (Fig. 6F). These findings implied that TASL expression may be a useful prognostic indicator for patients with “cold” tumor LGG and “hot” tumors LUAD and SKCM.

#### High TASL expression positively correlates with malignancy of gliomas in clinic

By analyzing gene expression data and clinical data from public databases, we found that TASL expression was higher in LGG and GBM tissues than in normal brain tissues (Figs. 1A and 6D), and high TASL expression was positively associated with shorter survival in glioma patients (Figs. 4E and 5), and high TASL expression was also a poor independent prognostic factor in LGG patients. Based on these, we further applied glioma cell lines and 71 glioma tissue samples to verify the reliability of the analysis results.

**Fig. 8:**
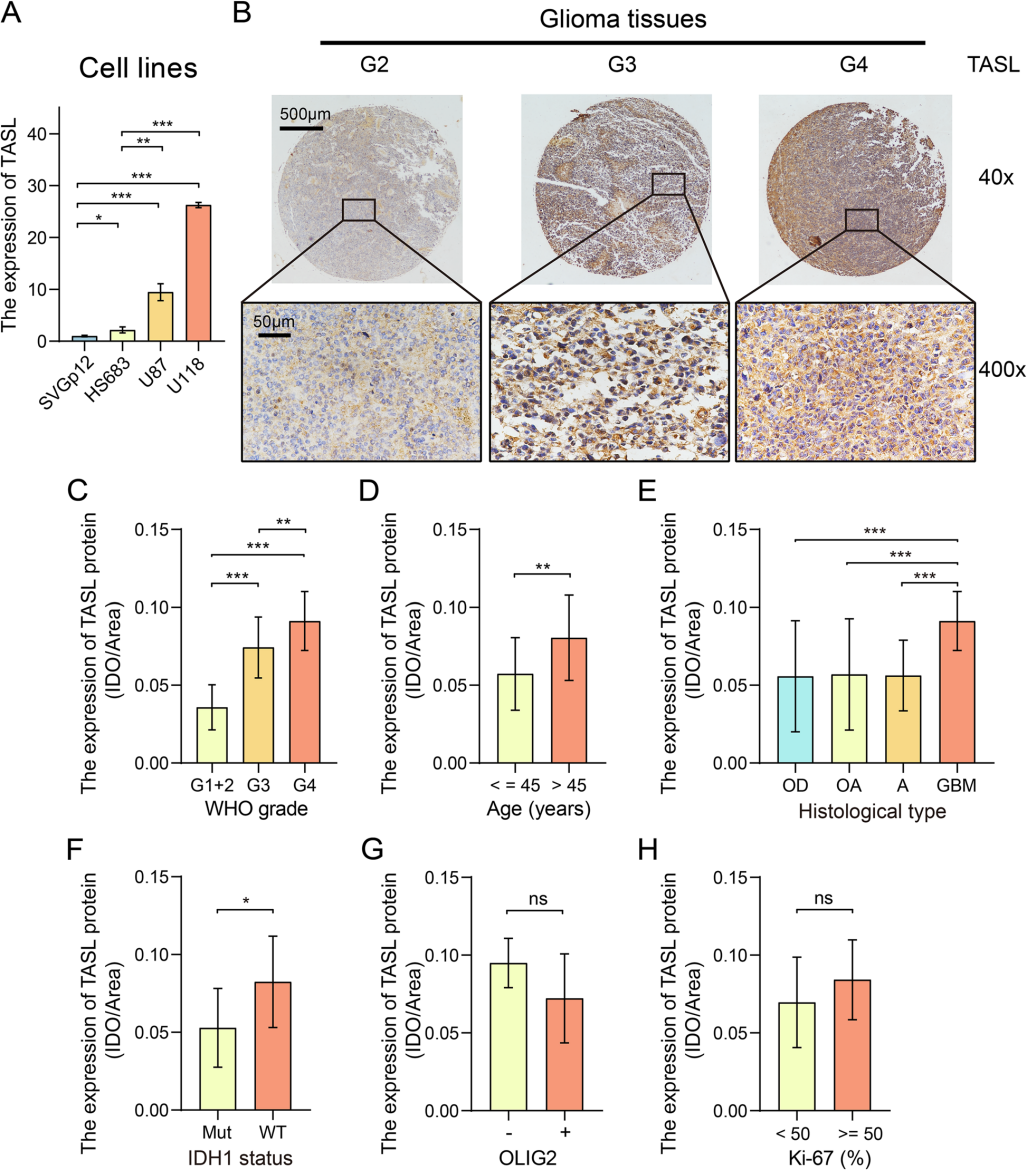
High expression of TASL positively correlates with poor clinicopathological features of glioma. **A** Validation of mRNA expression levels of TASL in cell lines (SVGp12, HS683, U87, and U118) using qRT-PCR. Representative images of IHC staining of TASL in pathological tissues of glioma patients of different WHO grades (40 × and 400x) (**B**) and statistical plots of TASL protein expression (**C**). TASL expression in glioma tissues grouped according to different age (**D**), pathological types (**E**), IDH1 mutation status (**F**), positive and negative status of OLIG2 expression (**G**) and positive rates of Ki-67 expression (**H**) (*P < 0.05; **P < 0.01; ***P < 0.001)

We first tested the mRNA expression of TASL in the human brain astrocyte line SVGp12, human LGG cell line HS683, and GBM cell lines U87 and U118 using qRT-PCR, and found that TASL expression was considerably higher in glioma cells than in normal astrocytes, and TASL mRNA expression was significantly higher in GBM cells than in LGG cells (*P* < 0.05, Fig. 8A). In addition, we tested the expression of TASL protein in human glioma tissues by IHC. We took images of representative samples of different WHO grades at 40 × and 400 × magnification and observed that TASL protein was mainly distributed in the cell membrane and cell plasma (Fig. 8B). We found that in gliomas, the expression of TASL increased with increasing WHO grade of patients (*P* < 0.01, Fig. 8C).

We also investigated the association of TASL protein expression with age, histological types, and clinical molecular indicators such as Ki-67 in glioma patients. TASL expression levels in tumor tissues of patients over 45 years of age were higher than those of patients 45 years of age and younger (*P* < 0.01, Fig. 8D); TASL expression levels in tumor tissues of patients with glioblastoma multiform were higher than those of patients with oligodendrogliomas, oligoastrocytomas, and astrocytomas (*P* < 0.001, Fig. 8E). TASL expression levels in tumor tissues of patients with IDH1-wildtype were higher than those of patients with IDH1 mutant patients (*P* < 0.05, Fig. 8F). As TASL expression increased, the expression of oligodendrocyte transcription factor 2 (OLIG2), a marker for identifying oligodendrocytes, marginally decreased in patients (*P* = 0.087, Fig. 8G), while the expression of the proliferation marker protein Ki-67 marginally increased (*P* = 0.068, Fig. 8H).

With the median TASL expression as the cut-off value, we divided the tumor samples into low and high TASL expression groups for analysis. The analysis showed that age (> 45 years), WHO grade (G4), pathological type (glioblastoma multiformes), and IDH1 mutation status (wild type) were statistically positively associated with high TASL expression (*P* < 0.05) (Table 2). The results of our clinical cohort study are consistent with those in public databases, suggesting that TASL expression in gliomas is positively correlated with the malignancy of the tumor.

**Table 2:** Correlation of TASL expression with clinicopathological parameters in 71 patients with glioma

Characteristics	TASL expression		P
	**Low (**%**)**	**High (%)**	
**Age (years)**			
< = 45	16 (22.5)	4 (5.6)	**0.003**
> 45	19 (26.8)	32 (45.1)	
**Gender**			
Male	22 (31)	19 (26.8)	0.536
Female	13 (18.3)	17 (23.9)	
**WHO grade**			
G1	1 (1.5)	0 (0)	** < 0.001**^a^
G2	14 (21.5)	0 (0)	
G3	9 (13.8)	8 (12.3)	
G4	8 (12.3)	25 (38.5)	
**Histological type**			
Oligodendrogliomas	4 (6.5)	2 (3.2)	**0.001**^a^
Oligoastrocytomas	5 (8.1)	2 (3.2)	
Astrocytoma	12 (19.4)	4 (6.5)	
Glioblastoma multiforme	8 (12.9)	25 (40.3)	
**IDH1 status**			
WT	10 (27.8)	17 (47.2)	**0.018**^a^
Mut	8 (22.2)	1 (2.8)	
**OLIG2**			
Negative	1 (1.7)	4 (6.8)	0.353^a^
Positive	28 (47.5)	26 (44.1)	
**Ki-67 positive (**%**)**			
< 50	26 (40)	21 (32.3)	0.190
> = 50	6 (9.2)	12 (18.5)	

^a^Denotes Fisher’s exact test

## Discussion

Even though ICIs have broken through the cancer treatment dilemma, immune “cold” tumors that lack response are still numerous. Targeting natural tumor immunity has emerged as a promising therapeutic direction. Increasing evidence suggests that the TLR pathway was associated with tumor development, prognosis, and treatment response, and acts as a “double-edged sword” to regulate tumor growth and function [33]. As an adaptor for TLR7-9 signaling, the TASL-SLC15A4 complex specifically activates the transcription factor IRF5 [23], which in turn induced the release of proinflammatory cytokines, chemokines, and IFNs [34], the latter of which can play an integral role in tumorigenesis and tumor progression. Therefore, TASL, a newly identified adaptor protein in the TLR pathway, may be a promising prognostic marker or immunotherapy target. However, there has been no study on the role of TASL in different cancers so far. We first analyzed TASL mRNA expression in tumor and normal samples of 33 cancer types and revealed a tissue-dependent role for TASL in tumor development. Some previous studies have also shown that, compared with normal tissues or cells, some types of tumor tissues or cells, such as HNSC [35], and SKCM [36], have the phenomenon of TLR pathway gene overexpression.

Immune escape and metabolic disorders are two major characteristics of cancer [37] and both of them were interdependent [38, 39]. In our study, differential enrichment of several immune, metabolic, and cancer-related pathways was observed between high and low TASL expression groups. TASL upregulates some immune-related pathways in most cancer types. Interestingly, a variety of classical metabolic pathways were extensively downregulated in the high TASL expression group, including oxidative phosphorylation, glutathione metabolism, etc. It is reasonable that oxidative phosphorylation is negatively correlated with immune pathways in cancer since reduced tumor immune activity is often accompanied by metabolic reprogramming from oxidative phosphorylation to glycolysis [28, 40]. There is evidence that TASL involves in regulating the endolysosome pH of immune cells such as dendritic cells and monocytes [41], while SLC15A4 (a proton-coupled amino acid transporter bound to TASL) is an important metabolic regulatory molecule [23], and the TLR pathway directly regulates metabolism and affects the behavior and function of tumors such as melanoma and breast cancer [42], which may provide strong evidence for our findings. Our results revealed that TASL may affect the occurrence and development of cancer by regulating immune and metabolic pathways, and TASL of the TLR pathway may be involved to some extent in the cross-talk between tumor immune and metabolic pathways. Furthermore, by analyzing the correlation between TASL expression and five immune signatures in 20 cancer types, we found the highest correlation between TASL expression and TILs. Numerous studies have shown that agonists of the TLR7-9 pathway promoted T-cell infiltration in TME [43, 44], which indirectly supported our results.

This is the first study to look into the impact of TASL expression on cancer survival. We found that high TASL expression may be an independent poor prognostic factor for the “cold” tumor LGG and an independent favorable prognostic factor for the “hot” tumors LUAD and SKCM. Several studies have found that increased TLR7 and TLR9 expression in glioma tissues is related to poorer prognosis [18, 45], while TASL is located downstream of TLR7-9, which indirectly supports the conclusion that high TASL expression is related to poor prognosis of LGG. Recently, it was reported that the TLR9 agonist PRINT-CpG was effective in promoting tumor regression in mouse models of non-small cell lung cancer [46], and the TLR7/8 agonist also has an antitumor effect in melanoma [47], which was consistent with this study.

The association with the TASL expression and immune-stimulating microenvironment in the “cold” tumor LGG, “hot” tumors LUAD and SKCM was revealed in this study. The higher expression of TASL was accompanied by a lower ratio of immunostimulatory to immunosuppressive cells in “cold” tumors and a higher ratio in “hot” tumors. We further comprehensively analyzed the correlation of TASL expression with the level of infiltration of various TIICs in the TME of LGG, LUAD, and SKCM, and the results implied that TASL may mediate the immune escape of the “cold” tumor LGG by inducing the conversion of Th1 cells to Th2 cells and the conversion of M1 macrophages to M2 macrophages. Conversely, TASL may enhance the ability of immune cells in the “hot” tumors LUAD and SKCM to kill tumor cells by recruiting Th1 cells in LUAD, CD8^+^ T cells in SKCM, and inducing the conversion of M2 macrophages to M1 macrophages in SKCM. In addition, activation of the TLR7-9 pathway has been found to transform the microenvironment of LUAD and SKCM from immunosuppressive to immune stimulating [43, 46]. These provided theoretical support for our results. These implied that TASL, a key factor in the TLR pathway, regulates opposite immune functions in the “cold” tumor LGG and the “hot” tumors LUAD and SKCM.

In addition, in “cold” tumor LGG, we also observed a significant negative correlation between TASL expression and CD56^bright^ NK cells and pDCs. CD56^bright^ NK cells have been shown to have antitumor activity [48], while pDCs promote innate and acquired immune responses through the production of IFN-ɑ, helping to induce and maintain antitumor immunity [49], which gives support to the speculation that LGG patients with high TASL expression have a lower survival rate possibly related to their negative regulation of antitumor immune activity. Intriguingly, we investigated TASL mRNA expression in different immune subtypes and found that the highest expressions of TASL were observed in the C4 subtype (lymphocyte depleted) of LGG, the C6 subtype (TGF-β dominant) of LUAD, and the C2 subtype (IFN-γ dominant) of SKCM. Thorsson V et al. revealed that tumor tissue with C4 subtype has low TIL level and high M2 macrophage infiltration, with poor prognosis. In contrast, the C6 subtype showed high TIL level, while the C2 subtype had the most abundant TIL level, strong CD8^+^ T cell signaling, and the highest M1 macrophage content, and had the most favorable prognosis [32], in striking agreement with our findings in LGG, LUAD, and SKCM. In conclusion, the correlation between TASL expression and the content of TIICs, in conjunction with the results of survival analysis, consistently suggested that TASL may contribute to the different prognosis of “cold” tumor LGG and “hot” tumors LUAD and SKCM, respectively, by influencing the infiltration of various TIICs in TME, especially TILs and TAMs.

A major bottleneck in current immunotherapy is the lack of appropriate biomarkers to predict therapeutic response. In recent years, several biomarkers have been reported, but their predictive performance has been unsatisfactory. Among them, PD-L1 protein expression has been identified as a predictive biomarker of anti-PD-1/PD-L1 treatment response in certain types of tumors. However, PD-L1 is ineffective in predicting “cold” tumors that lack immune cell infiltration [13, 50]. High TMB and high MSI are predictors of tumor response to immunotherapy because they can reflect tumor immunogenicity [51, 52]. However, new research suggested that they cannot predict ICIs response across all cancer types [12]. Based on this, the present study analyzed the predictive ability of TASL for tumor immune activity and response to immunotherapy. The results of the ROC curves showed that TASL was stronger than approved immunotherapy predictors such as TMB, MSI and PD-L1 in predicting immune score and TIL score, and the AUC values of the ROC curves for TASL to predict the response of cancer patients to immunotherapy ranged from 0.641 to 0.949, indicating that TASL can predict the immune activity of tumors and the efficacy of immunotherapy from some degree of accuracy.

Interestingly, we noticed that the high expression tends to correlate with longer OS and larger TIL fraction (this did not reach statistical significance, probably due to the small sample size) in the neoadjuvant anti-PD-1 with postoperative adjuvant anti-PD-1 (NA-aPD1) subgroup in analyzing the “cold” tumor GBM dataset GSE121810. While the high TASL expression was associated with shorter OS and not with TIL fraction in patients in the postoperative adjuvant anti-PD-1 (A-aPD1) subgroup. Since tumor samples from the NA-aPD1 subgroup were sequenced after surgical resection, we hypothesized that patients with high TASL expression who received neoadjuvant anti-PD-1 therapy had substantial activation of adaptive immunity in their TME and increased T-lymphocytes infiltration, giving the tumor a “hot” character, which allowed this subset of patients to be more responsive to ICIs in postoperative adjuvant therapy and have increased overall survival. Patients are more likely to respond to ICIs in postoperative adjuvant therapy and have longer overall survival. In contrast, in patients with high TASL expression who received only adjuvant anti-PD-1 therapy, the immune response in their TME was not well activated, the T lymphocyte infiltration was low, the tumors remained “cold”, and immunotherapy did not improve the clinical outcome of this group of patients. A previous study of the GSE121810 cohort also clearly showed that the addition of neoadjuvant anti-PD-1 therapy resulted in a broad and effective antitumor immune activation of the TME in gliomas compared to adjuvant anti-PD-1 therapy alone, resulting in prolonged survival [53].

To validate these results, we performed in vitro experiments using glioma cell lines and patient glioma tissues. qRT-PCR results showed that TASL expression was significantly higher in glioma cells than in normal astrocytes. IHC results indicated that the expression of TASL in tumor tissues of glioma patients was positively correlated with WHO grade. The results of the clinical cohort study were consistent with those in public databases, and TASL expression in gliomas was positively correlated with tumor malignancy, further suggesting the reliability of our analysis.

In our study, TASL has been inferred antitumor effect in SKCM and LUAD, but has a tumor-promoting effect in glioma, which may be related to the cellular composition of tumor tissues in different tumor types. We speculated that glioma, as an immune “cold” tumor with a small number of TIICs, was mainly composed of tumor cells. TASL might play a role in promoting cancer in tumor cells, while LUAD and SKCM were immune “hot” tumors, the extensive activation of TASL in immune cells leads to a better prognosis in patients with high TASL expression. Therefore, even with the important results of our correlation analysis, the mechanisms underlying the role of TASL in TME in different cancer types deserve further exploration.

## Conclusion

This study is the pioneer in systematically and comprehensively analyzing TASL in human cancers, and to some extent, elucidating the characteristics of TASL in different cancer types. TASL was significantly differentially expressed in different cancer types, with extensive heterogeneity at the transcriptional, genetic, and epigenetic levels. TASL has both suppressive and promotive effects on human cancer, and its high expression could be an independent poor prognostic factor for immune “cold” tumor LGG and a favorable prognostic factor for immune “hot” tumors LUAD and SKCM. Further analysis revealed that TASL may affect the level of tumor immune infiltration mainly by mediating TILs and TAMs, and TASL may differentially affect the prognosis of the three cancer types by mediating the immunosuppressive microenvironment in immune “cold” tumor LGG and the immunostimulatory microenvironment in immune “hot” tumors LUAD and SKCM. In addition, this study presents for the first time that high TASL expression is also a potential biomarker for the positive response to immunotherapy in various cancer types such as SKCM, which provides new ideas for current research in tumor immunotherapy.

## Methods

Supplementary Fig. 2 displays the detailed working flow of this study.

### Data collection and processing

RNA-seq data in transcripts per million reads (TPM) format for TCGA and GTEx were downloaded from UCSC XENA (https://xenabrowser.net/datapages/), then were processed and integrated uniformly by the Toil process (Supplementary Table 5), and RNA-seq data in R (3.6.3) for log2 transformation followed by TASL expression comparison between 33 tumor and normal samples. These 33 cancer types including Adrenocortical Carcinoma (ACC), Bladder Urothelial Carcinoma (BLCA), Breast Invasive Carcinoma (BRCA), Cervical Squamous Cell Carcinoma and Endocervical Adenocarcinoma (CESC), Cholangiocarcinoma (CHOL), Colon adenocarcinoma (COAD), Lymphoid Neoplasm Diffuse Large B-cell Lymphoma (DLBC), Esophageal Carcinoma (ESCA), Glioblastoma Multiforme (GBM), Head and Neck Squamous Cell Carcinoma (HNSC), Kidney Chromophobe (KICH), Kidney Renal Clear Cell Carcinoma (KIRC), Kidney Renal Papillary Cell Carcinoma (KIRP), Acute Myeloid Leukemia (LAML), LGG, Liver Hepatocellular Carcinoma (LIHC), LUAD, Lung Squamous Cell Carcinoma (LUSC), Mesothelioma (MESO), Ovarian Serous Cystadenocarcinoma (OV), Pancreatic Adenocarcinoma (PAAD), Pheochromocytoma and Paraganglioma (PCPG), Prostate Adenocarcinoma (PRAD), Rectum Adenocarcinoma (READ), Sarcoma (SARC), SKCM, Stomach Adenocarcinoma (STAD), Testicular Germ Cell Tumors (TGCT), Thyroid Carcinoma (THCA), Thymoma (THYM), Uterine Corpus Endometrial Carcinoma (UCEC), Uterine Carcinosarcoma (UCS) and Uveal Melanoma (UVM). RNA-seq data in HTSeq-FPKM format and related clinical data for 20 types of cancers were downloaded from TCGA (http://cancergenome.nih.gov/) and log2-transformed using R (3.6.3) for subsequent survival analysis and immune infiltration analysis.

The distribution of TASL mRNA in normal tissues was obtained from the Human Protein Atlas (HPA, https://www.proteinatlas.org/) database. Microarray expression profiling data for 20 types of cancers were downloaded from the GEO database (including GSE19750, GSE70947, GSE9750, GSE13898, GSE4290, GSE25099, GSE15641, GSE11151, GSE13159, GSE10072, GSE30219, GSE27651, GSE15471, GSE3325, GSE15605, GSE13861, GSE18155, GSE27155 and GSE2549). The expression of TASL in each dataset was normalized to [0, 1] using the formula: E_ normalized = [E-E_min_]/[E_max_-E_min_] (in which E_min_ and E_max_ stand for the minimal and maximal values of TASL expression in each gene set, respectively), and the E_ normalized values (Supplementary Table 6) were later applied to compare the TASL mRNA expression for each cancer type between cancer and normal tissues.

Gene expression profiles and clinical characteristics data were downloaded from three datasets from GEO (GSE72094, GSE65904, and GSE46517) and one dataset from the China Glioma Genome Atlas (CGGA, http://cgga.org.cn/) (mRNAseq_325) for the analysis of the association of TASL expression with survival prognosis in patients with LGG, LUAD and SKCM. In addition, gene expression profiles and clinical data were downloaded from Miao D et al. [29] and GEO (GSE19423, GSE111636, GSE67501, GSE100797, GSE91061 and GSE121810) were downloaded for the analysis of the association of TASL expression with response to cancer immunotherapy, as detailed in Table 1. The sources of all datasets were displayed in Supplementary Table 7.

This study also dealt with several gene sets representing various immune signatures: immune cytolytic activity (CYT) [54] cytokine and cytokine receptor (CCR) [55], human leukocyte antigen (HLA) [56], tumor-infiltrating lymphocyte (TIL) [57] and IFN [54], and 22 types of tumor-infiltrating immune cells (TIICs; including dendritic cells (DCs), Macrophages, natural killer cells (NKs), etc.) markers of the gene set [58]. Gene sets for other pathways were derived from the Kyoto Encyclopedia of Genes and Genomes (KEGG) [59–62] (Supplementary Table 8).

### Analysis of genetic alteration and DNA methylation

cBioPortal (https://www.cbioportal.org/) is an open data source that can analyze genomic alterations from a variety of cancer samples. We used the “TCGA Pan-Cancer Atlas Studies” module in cBioportal to query the genetic variant profile of TASL to determine the Copy Number Variants (CNVs), mutation type, and frequency of TASL. TMB measures the number of mutations in a given cancer genome, and we obtained somatic mutation data (MAF format) from the TCGA database and utilized the R package “maftools” to analyze and then calculate the TMB of patients. Microsatellite instability (MSI) was used to detect genetic instability in cancer, MSI data were obtained from Russell Bonneville et al. published in 2017 [63].

GSCA (http://bioinfo.life.hust.edu.cn/GSCA/) is a comprehensive database for genomic and immunogenomic cancer analysis. Thirty-three cancer types were selected with the help of the “Methylation” module to analyze the correlation between TASL mRNA expression and DNA methylation levels. Only the sites most negatively correlated with TASL expression were included in the analysis due to the presence of multiple methylation sites in a gene region usually.

### Survival analysis

COX regression analysis and Kaplan–Meier (K-M) curves were used to measure the association between TASL expression and overall survival (OS) in patients. R packages “survival” and “survminer” were used for statistical analysis. Based on the Maximum Selected Log-Rank Statistic, the “surv-cutpoint” function of “survminer” was used to find the optimum split point with the lowest P-value of Log-rank, and to divide patients into “high” or “low” TASL expression groups. Then plot the K-M curves, *P* < 0.05 were considered statistical significance.

### Immune infiltration-associated analysis

We used the CIBERSORT algorithm [64] to assess the levels of CD8^+^ T cells and regulatory T cells (Tregs) as well as M1 and M2 macrophages in various cancers. In addition, single sample gene set enrichment analysis (ssGSEA) [65] was used to estimate the infiltration levels of 22 TIICs in different cancer types. Differences in TASL mRNA expression in different immune subtypes were determined through the TISIDB website (http://cis.hku.hk/TISIDB/index.php/) which was used to study interactions between tumors and immunity. The ESTIMATE algorithm [66] was used to estimate stromal cell and immune cell scores for the quantification of the immune infiltration level of the tumor.

### Pathway enrichment analysis

We performed gene set enrichment analysis (GSEA) using the “clusterprofiler” package to further explore the KEGG pathways in which TASL is involved, |NES|≤ 1.0, *P* < 0.05 and False discovery rate (FDR) < 0.25 were deemed significant.

### Cell culture and quantitative real-time PCR (qRT-PCR)

Human normal astrocyte line SVGp12 and human GBM cell lines U87 and U118 (Cell Bank of Chinese Academy of Sciences, Shanghai, China), and LGG cell line HS683 (Procell, Wuhan, China) were cultured in DMEM medium (Biowest, FRA) containing 10% fetal bovine serum (HyClone, USA) at 37 °C and 5% CO_2_.

Total RNA was extracted from SVGp12, HS683, U87, and U118 cells using TRIzol (Invitrogen, USA). cDNA was synthesized using the PrimeScript™ RT Reagent kit (Takala, Japan). cDNA was extracted from SVGp12, HS683, U87 and U118 cells using THUNDERBIRD® SYBR® qPCR Mix (TOYOBO, Japan) for real-time PCR to detect TASL expression, and the experiments were repeated at least three times and calculated by the 2^−△△Ct^ method. The reaction conditions were as follows: 95 ℃ preheat for 5 min; 95 ℃ for 15 s, 57 ℃ for 30 s, 72 ℃ for 30 s, 5 cycles; 95 ℃ for 15 s, 59 ℃ for 30 s, 72 ℃ for 30 s, 35 cycles.

The following primers were used:

- TASL forward (5'-ACAAGTCAGAAGTCTCTACGTG-3');
- TASL reverse (5'-TCTCTCTGACTTCTGCTATGTTGG-3');
- GAPDH forward (5'-CCTGACCTGCCGTCTAGAAA-3');
- GAPDH reverse (5'-AGGAAAAGCAG-GAGGGTAGC-3').

### Tissues and immunohistochemistry (IHC)

With the approval of the Ethics Committee of China Medical University (approval No. CMU[2021]52), postoperative glioma tissues (*n* = 71) from the Department of Neurosurgery, Liaoning Cancer Hospital, China, from May 2017 to August 2019 were obtained for this study (Table 3).

**Table 3:** Basic information of 71 patients with glioma

Characteristics	n	(%)
**Age (years)**		
< = 45	20	28.2
> 45	51	71.8
**Gender**		
Male	41	57.7
Female	30	42.3
**WHO grade**		
G1	1	1.5
G2	14	21.5
G3	17	26.2
G4	33	50.8
**Histological type**		
Oligodendrogliomas	6	9.7
Oligoastrocytomas	7	11.3
Astrocytoma	16	25.8
Glioblastoma multiforme	33	53.2
**IDH1 status**		
WT	27	75
Mut	9	25
**OLIG2**		
Negative	5	8.5
Positive	54	91.5
**Ki-67 positive (**%**)**		
< 50	47	72.3
> = 50	18	27.7

Sections were obtained from formalin-fixed, paraffin-embedded brain glioma tissues. Sections were dewaxed and hydrated and placed in citrate buffer (pH = 6.0) for antigen retrieval, and endogenous peroxidase activity was blocked with 3% hydrogen peroxide. The slides were closed with 10% normal goat serum and incubated overnight at 4 °C with a 1: 100 dilution of the primary antibody (Rabbit anti-TASL antibody, ABclonal, China). The sections were stained with hematoxylin using horseradish peroxidase-labeled secondary antibody and 3, 3'-diaminobenzidine (DAB) as a substrate for color development.

Tissue sections were dried and placed under Nikon DS-Ri2 microscope for observation, and 5 representative images were randomly acquired in each cancer tissue using a 10 × eyepiece with a 40 × objective. The protein expression for each random field was quantified using Image Pro Plus (IPP) image processing software to obtain the average optical density values (sum of integrated optical density/area of interest (IOD/Area)), and then the average of IOD/Area of multiple fields was calculated to represent the protein expression of each tissue.

All quantitative data were tested for normality and homogeneity of variance. Student's t-test or Mann–Whitney U-test was used to determine the differences between continuous variables in the two groups. Pearson χ2 or Fisher's exact test was used to analyze the association between categorical variables and TASL expression in glioma patients. All statistical analyses and visualizations were performed in R (3.6.3), the R package “ggplot2”, and the Graphpad prism (8.0.2). *P* < 0.05 were considered statistical significance.

## Supplementary Information

**Additional file 1:** **Supplementary Figure 1.** Hierarchical survival analysis of the OS of LGG, LUAD and SKCM. (A) K-M curves of OS in LGG patients by WHO grade stratification. K-M curves of OS in LUAD (B) and SKCM (C) patients by AJCC T, N, M and pathologic stage stratification. **Supplementary Figure 2**. Overview of the study design. The study consisted of four main parts: I. Correlation of TASL mRNA expression with overall survival and clinicopathological parameters of patients; II. Correlation of TASL expression with immune infiltration signatures and tumor-infiltrating immune cell content in different cancer types; III. Correlation of TASL expression with immunotherapy response in tumor patients in the clinical setting; IV. Detection of TASL expression in glioma cell lines and clinical tissue samples using qRT-PCR and IHC.

**Additional file 2:** **Supplementary Table 1.** The NES values of KEGG pathways that significantly upregulated or downregulated in highly versus lowly TASL groups in more than five cancer types (|NES| ≥ 1.0, P<0.05, FDR<0.25). **Supplementary Table 2.** Correlation of TASL expression with immune infiltration signatures in 20 cancer types. **Supplementary Table 3.** ROC curves analysis of TASL, PDL1, PD1, CTLA4 expression, TMB, MSI, and glycolytic activity with immune score and TIL in 20 cancer types. **Supplementary Table 4.** Correlations of TASL expression with Immune checkpoints, TMB and MSI in 20 cancer types.**Supplementary Table 5.** Abbreviations, full names and sample sizes for 33 cancer types from the TCGA and GTEx databases. **Supplementary Table 6.** The original data (E_ normalized values) of TASL expression from GEO datasets used in Figure 6D. **Supplementary Table 7.** List of datasets from GEO, CGGA, and the article of Miao D, et al. involved in this study. **Supplementary Table 8.** List of gene sets.

## Data Availability

The datasets presented in this study can be found in online repositories. The names of the repositories and accession numbers can be found in the article/supplementary materials.
