# Supplementary material for: Pan-cancer analysis of TASL: a novel immune infiltration-related biomarker for tumor prognosis and immunotherapy response prediction
Source: BMC Cancer. 2023 Jun 9;23:528. doi: 10.1186/s12885-023-11015-w (PMC10251564; doi:10.1186/s12885-023-11015-w)
Supplement: Supplementary file 1 — Additional file 1: Supplementary Figure 1. Hierarchical survival analysis of the OS of LGG, LUAD and SKCM. (A) K-M curves of OS in LGG patients by WHO grade stratification. K-M curves of OS in LUAD (B) and SKCM (C) patients by AJCC T, N, M and pathologic stage stratification. Supplementary Figure 2. Overview of the study design. The study consisted of four main parts: I. Correlation of TASL mRNA expression with overall survival and clinicopathological parameters of patients; II. Correlation of TASL expression with immune infiltration signatures and tumor-infiltrating immune cell content in different cancer types; III. Correlation of TASL expression with immunotherapy response in tumor patients in the clinical setting; IV. Detection of TASL expression in glioma cell lines and clinical tissue samples using qRT-PCR and IHC. [file 12885_2023_11015_MOESM1_ESM.docx]

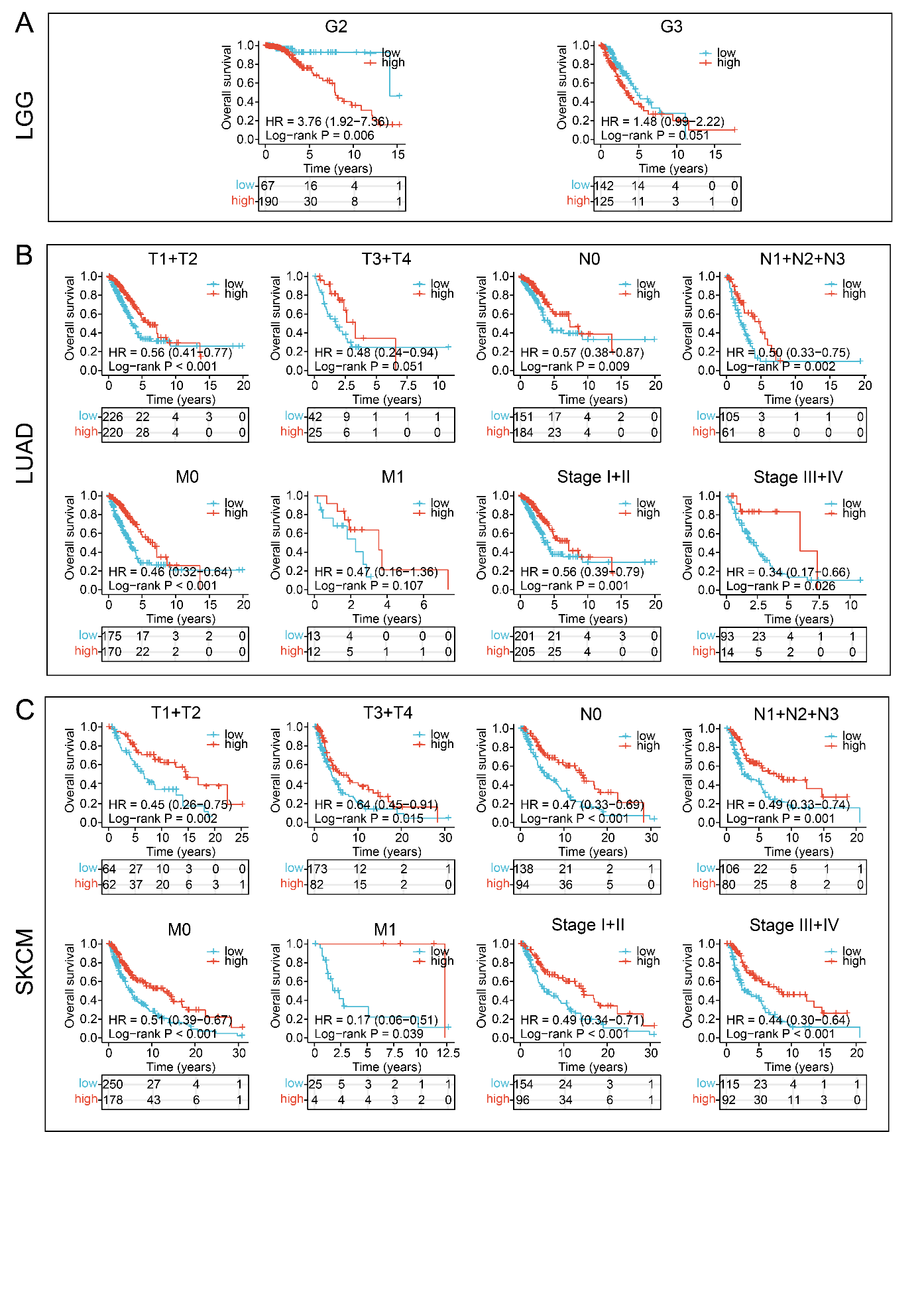


**Supplementary Figure 1**. Hierarchical survival analysis of the OS of LGG, LUAD and SKCM. (A) K-M curves of OS in LGG patients by WHO grade stratification. K-M curves of OS in LUAD (B) and SKCM (C) patients by AJCC T, N, M and pathologic stage stratification.


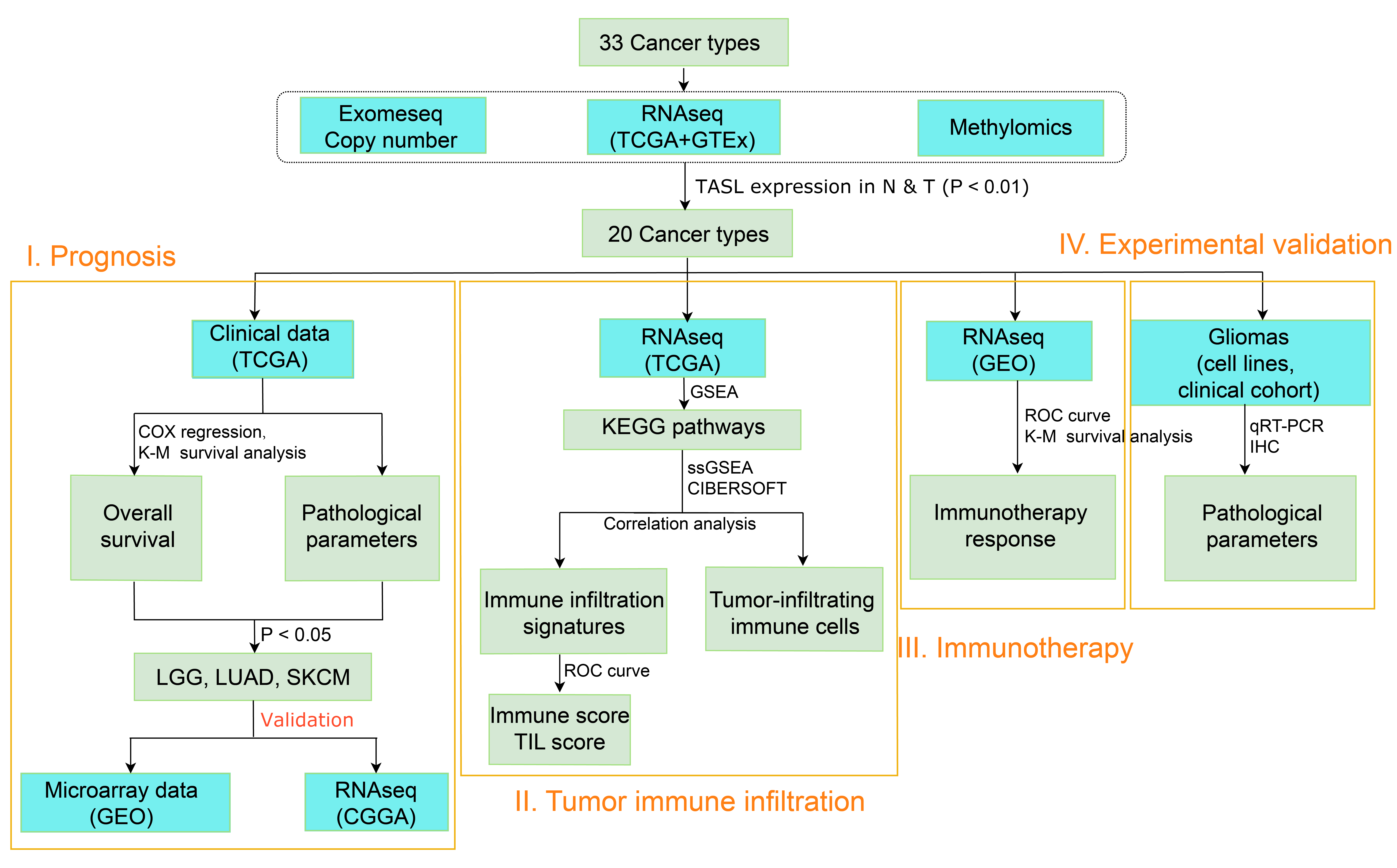


**Supplementary Figure 2.** Overview of the study design. The study consisted of four main parts: I. Correlation of TASL mRNA expression with overall survival and clinicopathological parameters of patients; II. Correlation of TASL expression with immune infiltration signatures and tumor-infiltrating immune cell content in different cancer types; Ⅲ. Correlation of TASL expression with immunotherapy response in tumor patients in the clinical setting; IV. Detection of TASL expression in glioma cell lines and clinical tissue samples using qRT-PCR and IHC.
